# Effect of CO_2_ Flow Rate on the Pinang Frond-Based Activated Carbon for Methylene Blue Removal

**DOI:** 10.1155/2013/545948

**Published:** 2013-08-21

**Authors:** S. G. Herawan, M. A. Ahmad, A. Putra, A. A. Yusof

**Affiliations:** Faculty of Mechanical Engineering, Universiti Teknikal Malaysia Melaka, Hang Tuah Jaya, 76100 Durian Tunggal, Melaka, Malaysia

## Abstract

Activated carbons are regularly used the treatment of dye wastewater. They can be produced from various organics materials having high level of carbon content. In this study, a novel Pinang frond activated carbon (PFAC) was produced at various CO_2_ flow rates in the range of 150–600 mL/min at activation temperature of 800°C for 3 hours. The optimum PFAC sample is found on CO_2_ flow rate of 300 mL/min which gives the highest BET surface area and pore volume of 958 m^2^/g and 0.5469 mL/g, respectively. This sample shows well-developed pore structure with high fixed carbon content of 79.74%. The removal of methylene blue (MB) by 95.8% for initial MB concentration of 50 mg/L and 72.6% for 500 mg/L is achieved via this sample. The PFAC is thus identified to be a suitable adsorbent for removing MB from aqueous solution.

## 1. Introduction

Agro waste pyrolysis is a method to convert biomass and organic residues into diverse products by heating in the absence of oxygen. Pyrolysis process is beneficial to understand the pyrolytic-cracking mechanism of a specific ligno-cellulosic waste [1] and it can be activated to produce activated carbon by subjecting a precursor to pyrolysis with oxidizing gases such as carbon dioxide (CO_2_) or steam (H_2_O) [2].

Activated carbon is an amorphous carbonaceous material which has a unique character exhibiting a high degree of porosity and extended interparticulate surface area. Activated carbon has an adsorption capacity for removing the undesirable odor, colour, taste, and both organic and inorganic impurities from domestic and industrial wastewater and for many more general domestic applications. Nearly 80% (~300,000 ton/year) of the total activated carbon is consumed for liquid-phase application in wastewater treatment [3].

Activated carbon can be produced from various organics materials having high carbon content like banana stalk [4], bamboo species [5], coffee husk [6], oil palm stone [7], date pits [8], oil palm shell [9, 10], and *Parkinsonia aculeata* wood [11]. This activated carbon can be produced using physical and chemical activation treatments. The advantages of physical activation are the possibility of developing more pores structure, large active area [8], and less effect from the secondary pollution problem during the disposal stage [12].

Pinang or *Areca catechu* in its scientific name is a tropical tree which belongs to *Arecaceae *family. It mainly grows from East Africa to the Arabian Peninsula across tropical Asia to the central Pacific and New Guinea. Traditionally, the main part used from this tree is the nut (or seed endosperm) where it is chewed as a stimulant masticatory. The practice can be found usually in India and some parts of Asia. Utilization of the frond is lacking and it is usually disposed [13]. In this paper, utilization of Pinang frond to produce an activated carbon is investigated. The utilization is expected to offer a potentially cheap alternative precursor.

In this research, Pinang frond was used as a precursor for preparing the Pinang frond-based activated carbon (PFAC) via CO_2_ physical activation. The effects of CO_2_ flow rate on the PFAC characteristic and its performance in removing the MB dye from aqueous solution are presented.

## 2. Material and Method

### 2.1. PFAC Preparation

The raw Pinang inang fronds were first collected from Kota Kuala Muda, Sungai Petani, Kedah, Malaysia. They were then cleaned and were subsequently dried at 110°C for 24 hours in the Heraeus Series 6000 Oven to remove the moisture content. The dried Pinang inang fronds were cut into pieces of dimensions around 5 × 4 cm and were loaded in a stainless steel vertical tubular reactor placed in a tube furnace with a programmable controller. A stainless steel wire mesh was placed at the bottom of the tubular reactor to prevent the sample from falling through. The ramp temperature was set to 20°C/min to achieve the activation temperature of 800°C under purified nitrogen (99.99%) at flow rate of 150 mL/min. The temperature within the sample bed in the reactor was measured by a K-type thermocouple. Once the activation temperature was reached, the activation agent of CO_2_ was introduced at various flow rates from 150 to 600 mL/min for 3 hours. The reactor was then cooled down to room temperature under nitrogen flow. The samples produced were stored in an airtight container for further characterization and adsorption studies. 

### 2.2. Characterization of PFAC

Characteristics of the samples were analyzed by using a surface area analyzer, a scanning electron microscopy (SEM), and a simultaneous thermogravimetric analyzer (STA). The surface areas of the samples were determined from the adsorption isotherms of nitrogen at 77 K by using Micromeritics ASAP 2020. The system was operated by measuring the quantity of gas absorbed on a solid surface at equilibrium vapor pressure by the static volumetric method. The calculations of the surface area, pore volume, and average pore size were performed by Micropore software version 2.46 whereas the specific surface area of the activated carbon was determined using the Brunauer-Emmett-Teller (BET) equation. The total pore volumes were estimated from the liquid volume of nitrogen at a relative pressure in the range of 0.01 to 0.98.

Scanning electron microscopy (SEM) was used to study the surface morphology of the samples including the pore structure, the surface structure, and the pore arrangement. The analysis was carried out using a Quanta 450 FEG SEM. The sample was put on the carbon tape on the aluminum stub and was coated with gold for electron conduction. The sample was then vacuumed for 5–10 min before analysis.

Proximate analyses of the samples were carried out using the PerkinElmer 6000 simultaneous thermogravimetric analyzer (STA). The STA system was interfaced to a microcomputer for data acquisition and control tasks. From the STA results, the moisture, volatile matter, fixed carbon, and ash contents can be obtained where each parameter is represented as a weight loss percentage from the total weight of the sample. The samples were heated from room temperature to 110°C in nitrogen N_2_ gas until dehydration was complete to obtain the moisture content. Decomposition was applied to the sample at 900°C to determine the amount of the volatile matter. Fixed carbon was obtained by switching the N_2_ flow to O_2_ flow. The remaining weight is represented as ash content. 

### 2.3. Methylene Blue

The methylene blue (MB) dye supplied by Sigma-Aldrich (M) Malaysia was used as an adsorbate. The MB has a chemical formula of C_16_H_18_ClN_3_S*·*3H_2_O with molecular weight of 373.9 g/mol. Deionized water was used to prepare all the reagents and solutions.

### 2.4. Batch Adsorption and Analysis System

Batch adsorption studies were carried out in a set of Erlenmeyer flask of 250 mL with 200 mL adsorbate solution of known initial concentration. Weight of the adsorbent was fixed at 0.2 g per flask. Isothermal water bath shaker was used at fixed 120 rpm at a constant temperature. The water bath shaker was equipped with a temperature controller which can be set from 25 to 100 ± 0.1°C and the rotation speed controller which can be fixed up to 250 ± 1 rpm.

A double-beam Shimadzu UV-Visible spectrophotometer was used to measure the concentration of the adsorbates. According to Beer's law, the linear relationship between absorbance and absorbing species concentration can be written as follows:

(1) $$\begin{array}{c} C=\frac{A_{i}}{\varepsilon \lambda bc}, \end{array}$$

where *C* is the solute concentration (mg/L), *A*_*i*_ is the measured absorbance for component *i*, *ελ* is the molar absorptivity coefficient of solute at wavelength *λ* (nm), and *bc* is the path length of the cell (1 cm). The absorbance *A*_*i*_ was obtained by the spectrophotometer through 1 cm path length of quartz cell.

The maximum wavelength of the MB was 664 nm. Calibration curve for MB dye concentration was measured to assure the homogeneity of the absorbance reading. The calibration curve for dye was obtained from the spectrophotometer as the plot of absorbance *A*_*i*_ against the solute concentration *C* for the percentage of dye removal can be calculated by

(2) $$\begin{array}{c} \%\;\;C=\frac{\left(C_{e}-C_{t}\right)}{C_{t}}\times 100,\; \end{array}$$

where *C*_*e*_ and *C*_*t*_ in mg/L are the liquid-phase concentrations of the adsorbate at equilibrium and at any arbitrary time *t*, respectively.

### 2.5. Preparation of Stock and Dye Solutions

Dye powder of 1.0 g was dissolved in 1000 mL of deionized water to prepare the concentration of 1 g/L dye solution. Solutions of different initial concentrations, that is, 50, 100, 200, 300, 400, and 500 mg/L, were prepared by dilution process of initial stock solution into 200 mL of deionized water.

## 3. Result and Discussion

### 3.1. Characterization of PFAC

Characterization of PFAC is important in order to determine the physical and chemical properties which affect the adsorption capacity. For convenience of discussion, the samples are labeled as in Table 1.

#### 3.1.1. Surface Area and Pore Volume

The effects of CO_2_ flow rate on PFAC in terms of surface area, pore volume, and average pore diameter were studied using surface area analyzer.  Table 2 shows that the PFAC_150_ is found to have the lowest BET surface area and total pore volume due to lack of CO_2_ flow rate supply which prevents optimum development of porosity.

Meanwhile, PFAC_300_ is found to have the highest BET surface area and total pore volume which are 958 m^2^/g and 0.5469 mL/g, respectively. This result shows that sufficient CO_2_ flow rate supply is required to create large specific surface area and high pore volume. However for PFAC_450_ and PFAC_600_ the surface area and total pore volume slightly decline compared to these for PFAC_300_. This is because the excessive CO_2_ flow rate which results in the wall of the pore structure to become thin and weak which then decreases the development of porosity. In terms of average pore diameter, all of the samples give the same result, that is, 2.32 nm diameter, signifying that the PFAC is in mesopore region accordance with the International Union of Pure and Applied Chemistry (IUPAC) classification [14].

#### 3.1.2. Pore Size Distribution

**Figure 1:**
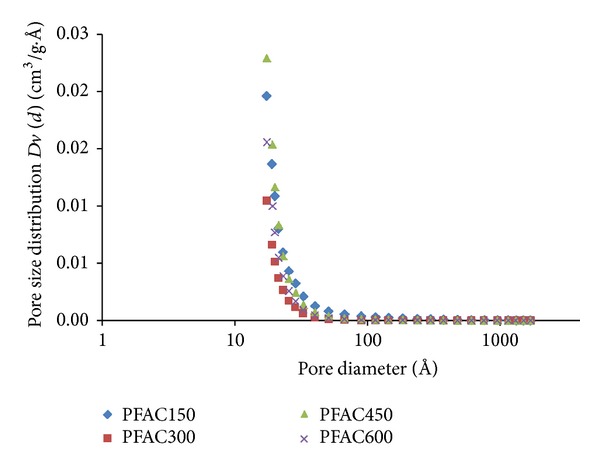
Pore size distribution of PFAC using BJH method.

Figure 1 shows the pore size distribution of the PFAC using the BJH method. As seen from the plot, singular sharp peak is detected in the range of 2-3 nm which is in the mesopore region. These results were consistent with those obtained for the average pore diameter. Mesopore surface area and mesopore volume of an activated carbon are the most important characteristics required for liquid adsorption, especially for removing dye [15].

#### 3.1.3. Surface Morphology

**Figure 2:**
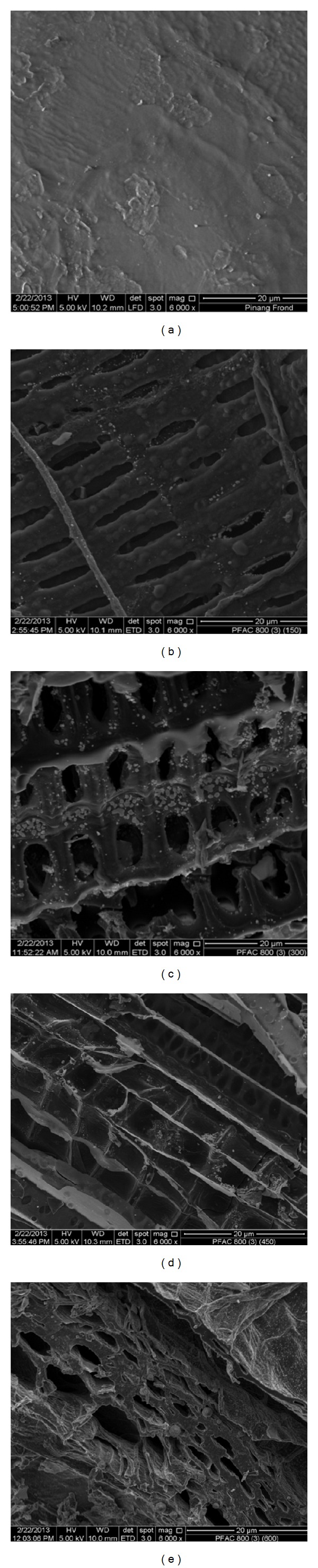
SEM images showing: (a) Pinang frond precursor, (b) PFAC_150_, (c) PFAC_300_, (d) PFAC_450_, and (e) PFAC_600_.

Figures 2(a)–2(e) show the surface morphology of Pinang frond and PFAC prepared at different CO_2_ flow rate conditions. It can be seen in Figure 2(a) that the surface texture of the Pinang inang frond precursor was rough, uneven, and undulating. Moreover very little pores were produced on the surface. However, after activation process, the PFAC generates some new pores which can be clearly observed in Figures 2(b)–2(e). In fact, during the activation treatment, the CO_2_ reaction occurred which promoted the pore development and hence increased the adsorption capacity and surface area of the sample [16].  Figure 2(b) for PFAC_150_ shows that the porosities have begun to develop. However, the porosity development does not look complete, having small opening of pores and thick wall. The sample of PFAC_300_ in Figure 2(c) shows the well-developed pore structure with larger pores and stable structured. For samples of PFAC_450_ and PFAC_600_ in Figures 2(d) and 2(e), the pore structures were unorganized and the structure wall begins to collapse.

#### 3.1.4. Proximate Analysis

The results for proximate analysis of the samples are tabulated in Table 3. The Pinang inang frond precursor was found to be high in volatile matter and moisture content. After activation process, the volatile matter and moisture content decrease significantly whereas the fixed carbon content increases in the sample. This condition occurs due to influence of pyrolytic effect at high temperature where most of the organic substances have been degraded and discharged both as gas and liquid tars resulting in a material with high carbon purity [17]. In proximate analysis, the main characteristic to be looked at is the fixed carbon content. Fixed carbon content plays an important role where the carbon surface holds the adsorbate molecules by the weak force known as Van Der Waals. Therefore, higher fixed carbon content means large carbon surface for the adsorbate to be adsorbed [18]. However, the existence of ash may sinter and block the pores which contributes significantly in decreasing the pores surface area [19]. PFAC_150_ to PFAC_600_ demonstrate similar result particularly for the fixed carbon and ash contents. The best result however is found for PFAC_300._

From the three previous analyses for the characterization of PFAC, PFAC_300_ gives the best result compared to other samples. From the surface area analyzer, PFAC_300_ has the highest value of the BET surface area and total pore volume. In the surface morphology study, PFAC_300_ is found to have wider opening pores as well as more organized and stable structure. Again from the proximate analysis, high fixed carbon with low ash content is also found on the sample PFAC_300_ and also the difference with other PFACs is not so significant_._ Therefore, PFAC_300_ sample is chosen for the subsequent studies.

### 3.2. Batch Adsorption Studies of Dye on PFAC

**Figure 3:**
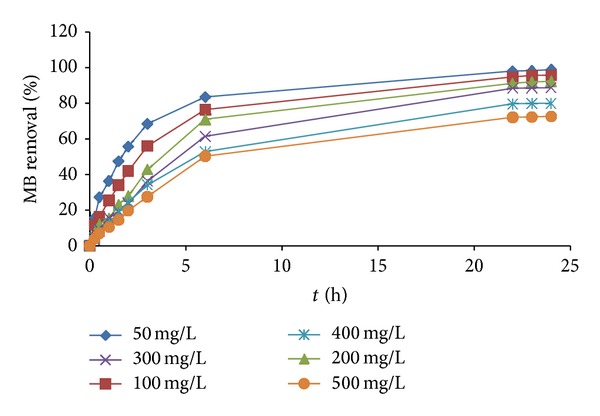
MB removal percentage versus adsorption time at various initial concentrations at 30°C for PFAC_300_.

Figure 3 illustrates the MB removal percentage versus the time interval obtained from (2). From the graph, it can be seen that the amount of the MB removal percentage increases with time and reaches a constant value above 20 hours. For the first 6 hours of batch adsorption, a rapid increase in MB removal can be observed for all MB concentrations. During the beginning of the adsorption process, rapid adsorption behavior occurs due to a powerful driving force from initial concentration that can overcome the mass transfer resistance between the aqueous and solid phases [20].

In the time interval of 6–20 hours, the MB removal slowly increases and reaches saturated above 20 hours. This condition is due to the fact that a large number of surface sites is available for adsorption at the initial stages and after a lapse of time, the remaining surface sites are difficult to be occupied because of the repulsion between the solute molecules of the solid and bulk phases [21]. In the end (24 hours), between 72.6% and 95.8% MB has been successfully removed at all initial dye concentrations where the latter is achieved using initial dye concentration of 50 mg/L. It can also be seen in Figure 3 that the removal percentage of dye is dependent upon initial concentration. Similar observation was also found in [22] on adsorption study of textile dyeing industrial effluent by flyash.

## 4. Conclusion

Pinang frond from waste precursor has successfully been utilized for the preparation of activated carbon using physical activation method. The optimum pyrolysis and activation agent CO_2_ flow rate of PFAC is found at 300 mL/min, where the BET surface area, the total pore volume, and the fixed carbon content are the highest compared to other CO_2_ flow rates. From the surface morphology, the PFAC_300_ creates well-developed pore structures with wider pores and stable structure. For the average pore diameter and the particle size distribution, PFAC structure is found in the mesopore region which is therefore suitable to be applied in a liquid phase adsorption. In batch adsorption study, PFAC is identified to be suitable for removing MB dye from aqueous solution. At equilibrium condition, PFAC_300_ is capable of adsorbing dye with removing 95.8% MB dye at initial concentration of 50 mg/L. Therefore, the preparation of activated carbon by applying correct amount of CO_2_ flow rate has been presented to be significant in developing the porosities and adsorption capability of the activated carbon.

## Figures and Tables

**Table 1:** List of label for the sample according to the process.

No.	Label	CO_2_ flow rate
1	PFAC_150_	150 mL/min
2	PFAC_300_	300 mL/min
3	PFAC_450_	450 mL/min
4	PFAC_600_	600 mL/min

**Table 2:** Surface area and pore characteristics of the PFAC.

Sample	CO_2_ flow rate (mL/min)	BET surface area (m^2^/g)	Total pore volume (mL/g)	Average pore diameter (nm)
Pinang frond	—	4	0.0087	7.20
PFAC_150_	150	847	0.4536	2.32
PFAC_300_	300	958	0.5469	2.32
PFAC_450_	450	936	0.5132	2.32
PFAC_600_	600	917	0.4909	2.32

**Table 3:** Result from proximate analysis.

Sample	Moisture (%)	Volatile (%)	Fixed carbon (%)	Ash (%)
Pinang frond	14.42	61.30	21.20	3.08
PFAC_150_	4.57	13.34	79.51	2.58
PFAC_300_	4.54	13.28	79.74	2.44
PFAC_450_	4.52	13.26	79.73	2.49
PFAC_600_	4.52	13.24	79.69	2.55
